# Differences between the neurogenic and proliferative abilities of Müller glia with stem cell characteristics and the ciliary epithelium from the adult human eye

**DOI:** 10.1016/j.exer.2011.09.015

**Published:** 2011-12

**Authors:** Bhairavi Bhatia, Hari Jayaram, Shweta Singhal, Megan F. Jones, G. Astrid Limb

**Affiliations:** Division of Ocular Biology and Therapeutics, UCL Institute of Ophthalmology and Moorfields Eye Hospital, 11 Bath Street, London EC1V 9EL, UK

**Keywords:** Müller glia, retina, ciliary epithelium, cell culture, retinal stem cells

## Abstract

Much controversy has arisen on the nature and sources of stem cells in the adult human retina. Whilst ciliary epithelium has been thought to constitute a source of neural stem cells, a population of Müller glia in the neural retina has also been shown to exhibit neurogenic characteristics. This study aimed to compare the neurogenic and proliferative abilities between these two major cell populations. It also examined whether differences exist between the pigmented and non-pigmented ciliary epithelium (CE) from the adult human eye. On this basis, Müller glia with stem cell characteristics and pigmented and non-pigmented CE were isolated from human neural retina and ciliary epithelium respectively. Expression of glial, epithelial and neural progenitor markers was examined in these cells following culture under adherent and non-adherent conditions and treatments to induce neural differentiation. Unlike pigmented CE which did not proliferate, non-pigmented CE cells exhibited limited proliferation *in vitro*, unless epidermal growth factor (EGF) was present in the culture medium to prolong their survival. In contrast, Müller glial stem cells (MSC) cultured as adherent monolayers reached confluence within a few weeks and continued to proliferative indefinitely in the absence of EGF. Both MSC and non-pigmented CE expressed markers of neural progenitors, including SOX2, PAX6, CHX10 and NOTCH. Nestin, a neural stem cell marker, was only expressed by MSC. Non-pigmented CE displayed epithelial morphology, limited photoreceptor gene expression and stained strongly for pigmented epithelial markers upon culture with neural differentiation factors. In contrast, MSC adopted neural morphology and expressed markers of retinal ganglion cells and photoreceptors when cultured under similar conditions.

This study provides the first demonstration that pigmented CE possess different proliferative abilities from non-pigmented CE. It also showed that although non-pigmented CE express genes of retinal progenitors, they do not differentiate into neurons *in vitro*, as that seen with Müller glia that proliferate indefinitely *in vitro* and that acquire markers of retinal neurons in culture under neural differentiation protocols. From these observations it is possible to suggest that Müller glia that express markers of neural progenitors and become spontaneously immortalized *in vitro* constitute a potential source of retinal neurons for transplantation studies and fulfil the characteristics of true stem cells due to their proliferative and neurogenic ability.

## Highlights

► Human pigmented ciliary epithelium does not proliferate or exhibit neurogenic properties *in vitro*. ► Non-pigmented ciliary epithelium proliferates *in vitro* and express markers of neural progenitors. ► Non-pigmented ciliary epithelium does not differentiate into retinal neurons *in vitro*. ► Human Müller glia that are highly proliferative express markers of neural progenitors *in vitro*. ► Müller glia that express neural progenitor markers differentiate into retinal neurons *in vitro*.

## 1 Introduction

Cells with characteristics of neural progenitor/stem cells have been isolated from the ciliary epithelium (CE) (Coles et al., 2004) and the neural retina (Klassen et al., 2004; Lawrence et al., 2007) of the adult human eye, and both cell populations have been considered as potential candidates for cell based therapies to replace or regenerate retinal neurons. Since the first report of the presence of CE with progenitor characteristics in the rodent retina and the subsequent identification of these cells in the adult human eye, extensive studies have been undertaken on the characterization and application of these cells to retinal transplantation using rodent models of retina degeneration. Several groups have reported that extended culture of CE causes their differentiation into progenitor-like cells (Ahmad et al., 2000; Fischer and Reh, 2003; MacNeil et al., 2007), leading to suggestions that these cells could be used to regenerate visual function. Despite the ciliary epithelium not being part of the neural retina, as a result of these reports CE cells were thought to constitute ‘retinal stem cells’ (Tropepe et al., 2000). However, despite the high volume of research in this field, no major advances in the use of CE cells for potential therapies have been so far achieved. On the contrary, more recent investigations have provided evidence that CE do not have the retinal neurogenic abilities previously thought. This is supported by findings that spheres formed by CE *in vitro* contain cells with epithelial properties and limited expression of neuronal markers as compared to neurospheres of neural stem cells (Moe et al., 2009). Furthermore, other reports have indicated that although cells isolated from the CE could be induced to express low levels of neuronal markers, they retained their epithelial morphology and failed to differentiate into retinal neurons (Cicero et al., 2009; Gualdoni et al., 2010), strongly suggesting that CE are not true retinal progenitor cells as previously suggested (Ahmad et al., 2000). Although recent studies suggest that human CE cells may have a functional capacity following transplantation *in vivo*, there is a lack of robust data to support this hypothesis (Inoue et al., 2009).

Studies in the zebrafish, known to regenerate retina throughout life, have demonstrated that Müller glia are able to de-differentiate into new retinal neurons *in vivo* (Raymond et al., 2006; Fimbel et al., 2007). Müller glia with stem cell characteristics have been identified in the postnatal chick (Fischer and Reh, 2001) and murine retina (Ooto et al., 2004; Karl et al., 2008), and more recent studies have shown that human Müller stem cells (hMSC) can also be isolated and expanded indefinitely *in vitro* from the adult human retina (Lawrence et al., 2007). In the light of recent studies that question the true progenicity of CE, at present, there are no comparative studies between pigmented and non-pigmented CE and hMSC in relation to their phenotypic features or their ability to differentiate into retinal neurons *in vitro*.

This study therefore aimed to compare pigmented and non-pigmented CE with Müller stem cells by examining their ability to survive and proliferate in culture and to express markers of neural progenitors and retinal neurons upon growth factors stimulation *in vitro*. Analysis of these features may aid in the understanding of the true regenerative potential of these two distinct cell populations within the human eye, and the targeting use of these cells as potential therapies to treat retinal degenerative disease, either by transplantation or endogenous induction of proliferation and neural differentiation *in situ*.

## 2 Materials and methods

### 2.1 Isolation of Müller cells with stem cell characteristics from the neural retina and of ciliary epithelial cells from the adult human eye

**Fig. 1:**
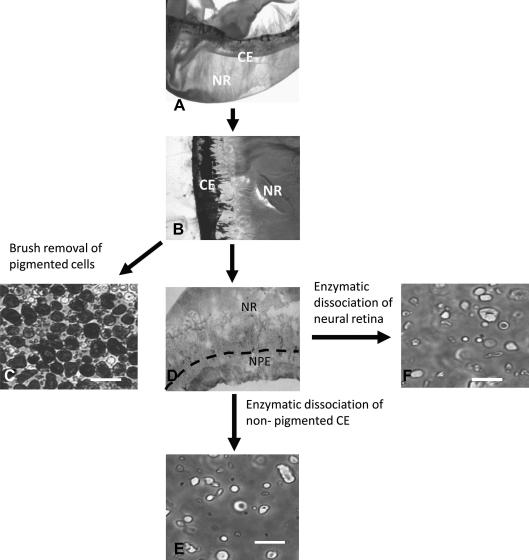
Isolation of stem cells from the human ciliary epithelium and neural retina. (A) The retina and ciliary body were detached from the choroid and RPE. (B) Pigmented cells were gently brushed away from the ciliary epithelium. (C) Microscopic appearance of pigmented CE cells upon isolation. (D) Broken line indicates the site from which non-pigmented ciliary epithelium was cut away from the neural retina. (E) Microscopic appearance of non-pigmented ciliary epithelial cells immediately after isolation. (F) Microscopic appearance of cells obtained from the neural retina immediately after isolation. (CE = ciliary epithelium, NPE = non-pigmented ciliary epithelium, NR = neural retina. Scale bars represent 20 μM.)

Acquisition of human tissue adhered to the tenets of the declaration of Helsinki. Following approval of the local research ethics committee, eyes with consent for research were obtained from Moorfields Hospital Eye Bank between 24 and 48 h post-mortem. After removal of the lens, retina and vitreous were gently dislodged from the eye cup using a pair of small forceps and placed in phosphate buffered saline (PBS). Müller stem cells were isolated as previously described (Limb et al., 2002) with some modifications to incorporate isolation of cells from the pigmented and non-pigmented ciliary epithelia. Using a dissecting microscope (Nikon SMZ1500, Nikon, Japan, http://www.nikon.com/), the pigmented ciliary epithelial cells were scraped away from the non-pigmented ciliary epithelium using a brush and scalpel and transferred to a tube containing PBS (Fig. 1). The non-pigmented ciliary epithelium was then separated from the neural retina. The non-pigmented ciliary epithelium and neural retina were individually transferred into different tubes containing PBS (Fig. 1). Dispase (Invitrogen, Paisley, U.K http://www.invitrogen.com) was added to reach a concentration of 2.4 U/ml and the tubes were incubated at 37 °C for 20 min. Hyaluronidase (at a final concentration of 0.67 mg/ml, Sigma–Aldrich, Gillingham, U.K, http://www.sigmaaldrich.com) and 10× Trypsin–EDTA (5% trypsin, 2% EDTA, Invitrogen) were then added to the dispase solution, the tissue was homogenised by vigorous pipetting and incubated for a further 20 min at 37 °C. Large tissue debris was then removed by filtration through a gauze mesh. Dissociated cells were pelleted by centrifugation and resuspended in the appropriate cell culture medium.

### 2.2 Culture of cells under adherent conditions

For adherent monolayer culture, isolated cells from the neural retina or ciliary epithelium were placed in 12.5 cm^2^ tissue culture flasks (Becton, Dickinson and Company (BD Biosciences http://www.bdbiosciences.com) coated with 10 μg/cm^2^ fibronectin (Sigma) in the presence of 40 ng/ml of epidermal growth factor (EGF) in DMEM medium containing GlutaMAX I (Invitrogen) and 10% foetal bovine serum (FBS) (Invitrogen). The original medium was maintained for one week and then replaced weekly. Colonies formed after 2–3 weeks, cells were detached by incubation with Trypsin–EDTA for 3 min at 37 °C and resuspended in fresh media before plating. When cells reached confluence, they were plated at a concentration of 2.5 × 10^5^ cells/ml and cultured with or without the addition of EGF to the culture medium.

### 2.3 Culture of cells under non-adherent conditions

Cells isolated from the neural retina and ciliary epithelium were suspended in DMEM medium containing GlutaMAX I, 40 ng/ml of EGF and B-27 supplement without Vitamin A (Invitrogen). The medium was replaced every two days. After a week culture in suspension, cells were transferred to adherent culture conditions (as indicated in the previous section) and passaged when confluent. Cells were otherwise passaged weekly and were plated at a dilution of 10^4^ cells/ml with the addition of EGF to the culture medium.

### 2.4 Assessment of cell proliferation

Following detachment with Trypsin/EDTA and prior to replating, cells were pooled in 1–2 ml of medium and counted in a Neubauer haemocytometer using the trypan blue exclusion method. Cells were cultured at a concentration of 1 × 10^4^ cells per well in 6 well plates. The total number of cells was calculated by multiplying the initial number of cells by the number of divisions obtained after each passage. The number of divisions was calculated by dividing the total number of cells obtained at each passage by the number of cells plated. The resulting number was plotted to analyse the total number of cells obtained from each cell preparation.

### 2.5 Neural differentiation *in vitro*

For differentiation studies, cells were plated onto flasks coated with basement membrane protein (ECM gel from Engelbreth-Holm-Swarm murine sarcoma, Sigma) at a concentration of 10^4^ cells/ml. Cells were cultured in DMEM medium containing 5% FCS, fibroblast growth factor 2 (FGF2/bFGF-40 ng/ml, Sigma) and DAPT (N-[N-(3,5-Difluorophenacetyl)-l-alanyl]-S-phenylglycine t-butyl ester, Sigma) diluted in DMSO to a concentration of 50 μM/ml. Media was replaced every 3 days and cells were cultured for a total of 7 days.

### 2.6 Immunostaining of cells

Cells were fixed in 4% Paraformaldehyde for 10 min and cryoprotected with 30% sucrose. Slides were blocked for 1 h in 0.5% Blocking Solution (Roche, U.K. http://www.roche-applied-science.com). Primary antibodies were diluted in the blocking reagent and incubated with the cells overnight at 4 °C. The primary antibodies used were: CD44 (F10-44-2; monoclonal; 1:1000; AbD Serotec, Oxford, U.K., http://www.abdserotec.com), CHX10 (SC-21692; goat; 1:200; Santa Cruz, U.S.A. http://www.scbt.com), Nestin (MAB5362; monoclonal; 1:1000; Chemicon (Millipore), Billerica, U.S.A. http://www.millipore.com/), RPE65 (ab13826; monoclonal; 1:200; Abcam, Cambridge U.K http://www.abcam.com), Pan-cytokeratin (MAB3406; 1:200; monoclonal; Chemicon (Millipore)), SHH (SC-9024; rabbit; 1:200; Santa Cruz), SOX2 (AB5603; rabbit; 1:1000; Chemicon (Millipore), Vimentin (SC-130610; 1:500; rabbit; Santa Cruz). Specific binding of primary antibodies was detected using donkey anti-IgG labelled with Alexa Fluor 488 or 555 (Molecular Probes, Invitrogen) reacting with the species in which the primary antibody was raised for 1 h at room temperature, before incubating with DAPI (Sigma) to visualise cell nuclei and mounted using Vectashield (Vector Laboratories, U.S.A. http://www.vectorlabs.com). Fluorescent images were recorded using confocal microscopy (Leica TCS SP2 AOBS, Leica, Germany http://www.leica-microsystems.com) using a 40× oil objective. Images were analysed using the Leica Confocal software.

### 2.7 RT-PCR

Total RNA was extracted using the RNeasy kit (Qiagen http://www.qiagen.com) according to the manufacturer’s instructions. RNA (1 μg) was transcribed into cDNA using AMV Reverse Transcriptase (Roche). The reaction was performed in a final volume of 20 μl consisting of 5 mM MgCl_2_, 1 mM dNTP, 1 U/μl RNase inhibitor, 0.8 U/μl AMV reverse transcriptase and 80 ng/μl oligo dT-15 primers. The mixture was incubated for 10 min at 25 °C, 60 min at 42 °C, 5 min at 99 °C and 5 min at 4 °C in a thermal cycler (Eppendorf, http://www.eppendorf.co.uk). The cDNA (5 μl) was used for PCR reactions using the High Fidelity PCR kit (Roche). The amplification was performed in a final reaction volume of 25 μl consisting of 1.5 mM MgCl_2_, 0.2 mM dNTP, 2.5 U Expand HiFi Taq DNA polymerase, 0.4 μM primers in 50 mM KCL (see Table 1 for primer sequences), 10 mM Tris/HCl, pH 8.0. The mixture was incubated at 94 °C for 2 min followed by 30 cycles under the following conditions: 94 °C for 30 s, 60 °C for 30 s, 72 °C for 1 min and 1 cycle of 72 °C for 5 min. An annealing temperature of 60 °C was used for all primers except for RPE65 where a temperature of 55 °C was used. Products were run on 1% agarose gel containing 1 in 15,000 dilution of Sybr Gold (Invitrogen). GAPDH primers were used as an internal control.

### 2.8 Western blotting

Cell lysates were extracted using Radio Immuno Precipitation Assay (RIPA) buffer containing a protease inhibitor cocktail (Sigma). Protein concentration was estimated using the Bradford assay system (Bio-Rad, Hemel Hempstead, U.K. www.biorad.com). Protein volumes loaded onto the electophoresis gel were adjusted according to the relative absorbance readings. In addition a loading control (β-actin) was also used in each experiment. The NuPAGE gels and buffer systems from Invitrogen were used for Western Blots. 30 μl of loading sample was prepared with 3 μl of reducing agent (10×), 7.5 μl of loading buffer (LDS 4×) and a maximum of 19.5 μl of protein sample. The loading samples were boiled at 80 °C for 10 min to denature the protein. The samples and protein ladder were loaded onto 10% bis-tris gels with MOPS buffer containing antioxidant and were run at 180 V for 50 min. The proteins were transferred onto Polyvinylidene Fluoride (PVDF) membranes (Hybond-P, Amersham, Little Chalfont, U.K. http://www.gelifesciences.com) at 35 V for 90 min. Membranes were blocked in Tris Buffered Saline with 0.1% Tween-20, 5% skimmed milk and 3% FBS at 37 °C for 1 h. Primary antibodies (BRN3B (N15; SC-31987; 1:200; goat; Santa Cruz) and β-actin (1:5000; monoclonal; Sigma)) were diluted in blocking buffer. Membranes were incubated overnight at 4 °C with the primary antibodies, before incubation with a secondary antibody conjugated with Horseradish peroxidase (1:10,000, Jackson Laboratories http://www.jacksonimmuno.com) for 1 h at room temperature. Blots were visualised by chemiluminescence using ECL advanced detection reagent (GE Healthcare http://www.gelifesciences.com) and a Fujifilm LAS-100 imager (www.fujifilm.com).

## 3 Results

### 3.1 In vitro characteristics of Müller glial stem cells and ciliary epithelium cultured under non-adherent conditions upon isolation

In previous studies, ciliary epithelium (CE) derived progenitor cells have been cultured in suspension using the B27 supplement and serum-free media for 1 week before being transferred to adherent culture conditions to generate cell lines (Coles et al., 2004). Human Müller glia isolated from the neural retina that become spontaneously immortalized and that express stem cell markers are normally cultured as monolayers immediately after isolation in the presence of FBS (Lawrence et al., 2007).

**Fig. 2:**
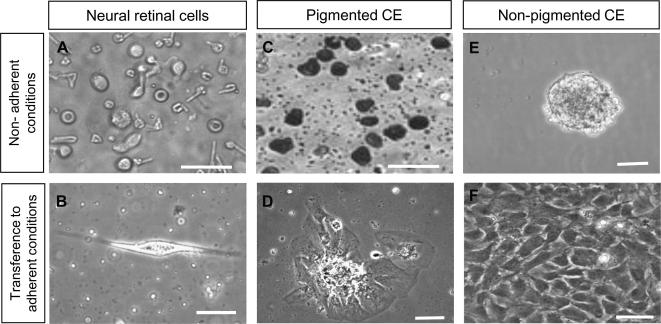
Phase-contrast microscopy of cells isolated from the neural retina and CE cultured in suspension for 1 week before transferal to adherent culture conditions (A) Cells isolated from the neural retina did not form spheres when cultured in suspension. (B) Neural retinal cells were able to adhere to tissue culture plates after initial culture in suspension for 1 week. However, these cells did not proliferate. (C) Pigmented CE cells did not adhere after 72 h in suspension culture. (D) When transferred to adherent conditions, pigmented CE cells attach, lost their pigment and acquired a large flattened/epithelioid morphology and did not proliferate. (E) Non-pigmented CE cells formed spheres after 72 h in suspension culture. (F) When cultured under adherent conditions in the presence of EGF, dissociated CE cells from non-pigmented spheres reached confluency by 1 week and displayed a characteristic epithelial morphology. (Scale bars represent 100 μm. CE = Ciliary Epithelium, EGF = Epidermal Growth Factor.)

When cultured under non-adherent conditions in the absence of serum and but in the presence of B27 supplement and EGF, Müller glia isolated from the neural retina did not form spheres (Fig. 2A). After one week culture in suspension, retinal Müller cells were transferred to adherent conditions in the presence of 10% FBS and EGF. Under these conditions a few cells attached to the plate and displayed an elongated morphology (Fig. 1B) but did not proliferate. When cultured in suspension, primary cells obtained from the pigmented CE did not adhere or form spheres (Fig. 2C), whilst non-pigmented CE cells formed spheres within 72 h of culture (Fig. 2E). When transferred to adherent conditions, cells from the pigmented CE adhered to the plates and exhibited a flattened and aberrant epithelial morphology suggestive of terminal differentiation (Fig. 2D). In contrast, cells derived from spheres formed by non-pigmented CE cells, when transferred to adherent cultures exhibited a characteristic epithelial morphology (Fig. 2F), proliferated and rapidly reached confluence. These cells continued proliferating in the presence of EGF for up to 20 passages examined.

### 3.2 In vitro characteristics of Müller glial stem cells and ciliary epithelium cultured under adherent conditions upon isolation

**Fig. 3:**
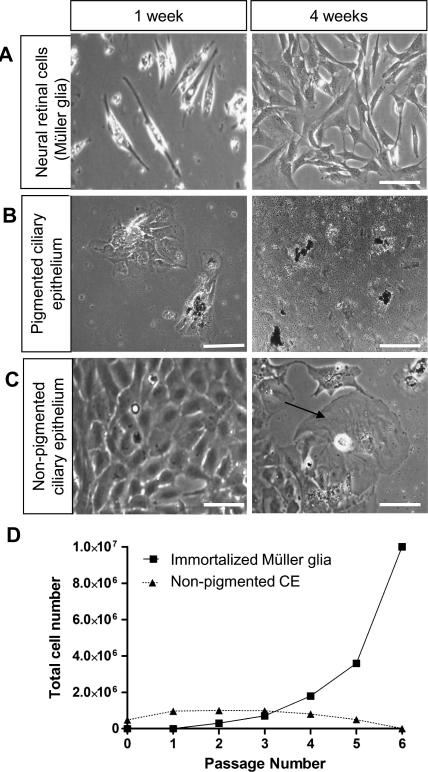
Phase-contrast microscopy and proliferation of Müller stem cells and CE cells cultured under adherent conditions in the presence of EGF (A) (i) A small number of phase bright cells isolated from the neural retina were observed attached to the plate one week after isolation, (ii) small colonies of these cells displaying a characteristic glial morphology were observed after 2–3 weeks in culture. (B) (i) Pigmented CE cells showed an aberrant epithelioid morphology after 1 week in culture and did not proliferate. (ii) After 4 weeks in culture, pigmented CE cells were still present but did not proliferate. (C) (i) After 1 week in culture, non-pigmented CE cells had adhered and proliferated. Confluent monolayers exhibited characteristic epithelial morphology at 1 week. (ii) After 4 weeks culture, the rate of proliferation of these cells had decreased and many of them showed aberrant morphology, becoming squamous and showing visible actin filaments (arrows). (D) Non-pigmented CE initially proliferated at a faster rate than those derived from the neural retina. However by passage 6, the majority of cells had died. Cells derived from the neural retina that became spontaneously immortal and later showed stem cell characteristics did not proliferate until after 2–4 weeks in culture, but continued to grow indefinitely in the absence of EGF. (Scale bars represent 100 μm. CE = Ciliary Epithelium, EGF = Epidermal Growth Factor.)

Primary cells isolated from the neural retina, when cultured with medium which contains 10% FBS and EGF, adhered readily to tissue culture plates coated with fibronectin. Most cells isolated from the neural retina that adhered to the plates appeared bright when viewed under a phase objective, and displayed a characteristic glial morphology after 1 week in culture (Fig. 3A). Colonies of proliferating Müller glia appeared after 2–3 weeks in culture under adherent conditions and could be dissociated and further expanded upon removal of the EGF initially added to the primary cultures (Fig. 3A). Müller cells that displayed these features became spontaneously immortalized and exhibited characteristics of stem cells as previously described (Lawrence et al., 2007).

Pigmented and non-pigmented cells from the CE cultured on fibronectin coated plates in the presence of FBS and EGF also attached readily. After 1 and 4 weeks in culture, pigmented CE cells that had originally adhered to the plate did not proliferate (Fig. 3B). In contrast, non-pigmented CE cells divided rapidly and showed confluence within 1 week (Fig. 3C). During the first 3 weeks in culture, non-pigmented CE divided more rapidly than Müller glia with stem cell characteristics (Fig. 3D), but by 4 weeks in culture a large proportion of them had acquired a squamous aberrant morphology with visible stress fibres (Fig. 3C) and stopped proliferating despite the presence of EGF (Fig. 3D). Comparison of the cell yields obtained over successive passages of both Müller glia and non-pigmented CE cells cultured under adherent conditions in the absence of EGF showed that although non-pigmented CE cells proliferated soon after isolation, over successive passages they declined in number and by passage 6 they had stopped proliferating. In contrast, Müller glial cells did not start proliferating until 2–3 weeks in culture, but they grew exponentially and continued to proliferate indefinitely to become spontaneously immortalized (Fig. 3D).

### 3.3 Comparison between the expression of epithelial and glial markers by spontaneously immortalized Müller glia and non-pigmented CE cells

**Fig. 4:**
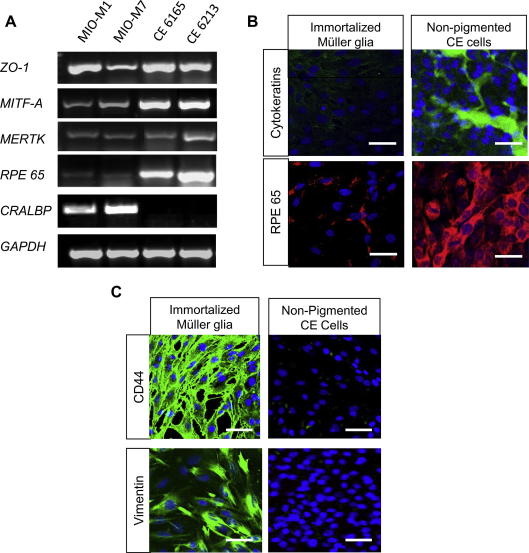
Comparison between the expression of glial and epithelial cell markers by immortalized Müller glia with stem cell characteristics and non-pigmented CE cells (A) RT-PCR analysis of Müller (MIO-M1 and MIO-M7) and non-pigmented CE cells (CE 6165 and CE 6213) for mRNA expression of retinal pigment epithelial (RPE) markers showed that both cell types expressed mRNA for the RPE cell markers ZO-1, RPE65, MITF-A and MERTK. However, non-pigmented CE cells showed higher expression of these markers than Müller stem cells. mRNA coding for CRALBP, a characteristic marker of Müller glia was expressed by Müller cells but not by CE cells. GAPDH primers were used as control. (B) Immunostaining of Müller and non-pigmented CE cells for markers of RPE. (i) Non-pigmented CE cells showed strong staining with a pan-cytokeratin antibody, whilst no expression of this marker was observed in Müller stem cells. (ii) A small proportion of Müller stem cells stained for RPE65 (less than 10%), whilst all CE cells stained strongly for this protein. (C) Staining for glial markers showed that Müller cells that expressed stem cell properties expressed the proteins CD44 and vimentin, whilst CE cells did not stain for these markers. (CE = Ciliary Epithelium, EGF = Epidermal Growth Factor. Scale bars represents 50 μm.)

Both spontaneously immortalized Müller and non-pigmented CE cells expressed mRNA coding for genes expressed by retinal pigment epithelium (RPE), although stronger expression of markers of RPE cells was observed in CE cells when compared with immortal Müller glia (Fig. 4A). These included the tight junction marker *ZO-1*, *MITF-A* (microphthalmia-associated transcription factor A, a protein highly expressed in RPE), *MERTK* (a receptor tyrosine kinase involved in phagocytosis of photoreceptor outer segments by RPE) and *RPE65* (an enzyme involved in 11-cis retinal synthesis). In contrast with the marked expression of cellular retinaldehyde binding protein (CRALBP) by Müller cells, non-pigmented CE cells did not express this molecule. The high mRNA expression of RPE cell markers in CE cells and their lack of expression of CRALBP, one of the main Müller glial cell markers, highlights the pigmented epithelial origin of these cells.

Immunostaining for RPE65 and cytokeratins by CE further confirmed the retinal epithelial nature of the cultured non-pigmented CE cells. In contrast, hMSC did not stain for cytokeratins and only a small number of these cells showed a weak staining for *RPE65* (Fig. 4B).

Staining for glial proteins also revealed differences between the two cell populations. Immortalized Müller glia strongly stained for the hyaluronan receptor CD44 and the glial protein Vimentin, both of which are expressed by Müller glia *in situ* (Nishina et al., 1997), contrasting with the lack of glial marker expression by non-pigmented CE cells (Fig. 4C).

### 3.4 Expression of neural stem cell markers by Müller glia spontaneously immortalized and non-pigmented CE in culture

**Fig. 5:**
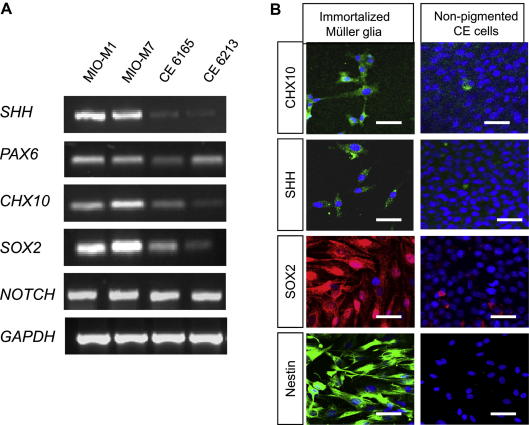
Expression of stem cell markers by Müller glia and non-pigmented CE (A) RT-PCR analysis of two Müller stem cell lines that became spontaneously immortal (MIO-M1 and MIO-M7) and two non-pigmented CE cell preparations (CE 6165 and CE 6213). All cells expressed the retinal progenitor markers *SHH*, *PAX6*, *CHX10*, *SOX2* and *NOTCH1*. However, a lower expression of *SOX2* was observed in non-pigmented CE cells, when compared with Müller stem cells. *GAPDH* primers were used as control. (B) Immunostaining of Müller and non-pigmented CE cells for markers of retinal progenitors. A proportion of Müller stem cells showed expression of CHX10, SHH and SOX2, whilst non-pigmented CE cells did not stain positively for these proteins. Müller cells with stem cell characteristics also expressed the intermediate filament protein Nestin, a marker of neural stem cells, whilst CE cells did not express this protein. (CE = Ciliary Epithelium, EGF = Epidermal Growth Factor. Scale bars represents 50 μm.)

Müller glia that spontaneously became immortalized and non-pigmented CE cell lines grown in the presence of EGF expressed markers of retinal progenitors as determined by RT-PCR analysis (Fig. 5A). Both cell types expressed mRNA coding for *SHH*, *PAX6*, *CHX10*, *SOX2* and *NOTCH1*. The intensity of expression of *CHX10*, *SOX2* and *PAX6* amplicons appeared qualitatively lower in CE cells when compared to spontaneously immortalized Müller glia.

Immunostaining confirmed the protein expression of progenitor markers in both immortalized Müller glia and non-pigmented CE cells. The majority of immortalized Müller glia stained for the retinal progenitor markers CHX10, SHH and SOX2 (Fig. 5B). Staining for CHX10 and SOX2 was seen in both the cytoplasm and nucleus, and predominant nuclear staining for SOX2 was characteristically observed in immortalized Müller cells. These cells also stained for SHH, supporting suggestions that Müller glia may have the potential to utilize this signalling pathway towards neurogenesis (Wan et al., 2007). Müller stem cells also stained intensely for the intermediate filament protein Nestin, a marker of neural stem cells (Fig. 5B). Only a very small number of CE cells (1–2%) stained for CHX10, SHH and SOX2, but expression of Nestin was not observed in CE cells at any time during culture.

### 3.5 Neural differentiation of spontaneously immortalized Müller glia and CE cells

**Fig. 6:**
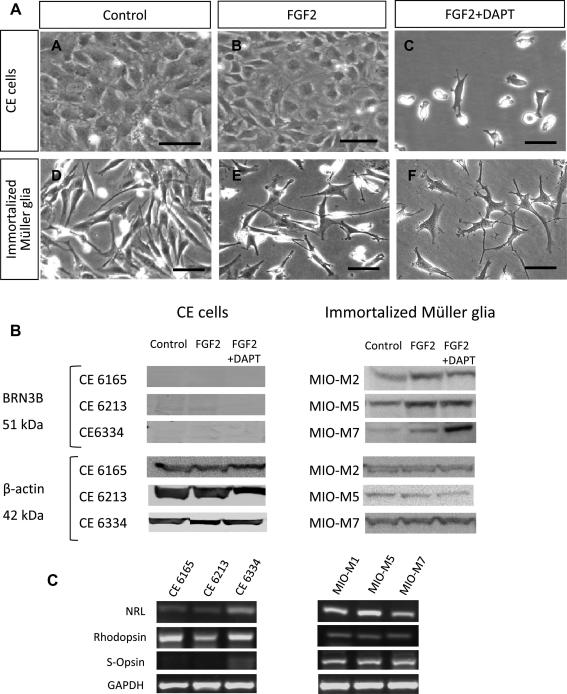
Neurogenic ability of non-pigmented CE and Müller stem cells cultured under differentiating conditions for 7 days (A) (i) Morphology of CE and Müller cells cultured under normal culture conditions in the presence of 10% serum. Non-pigmented CE cells were maintained with EGF. (ii) Under adherent conditions in the presence of ECM gel and FGF2, non-pigmented CE cells maintained their epithelial cell morphology, however, a few flattened cells were visible. In contrast, Müller stem cells acquired a neural-like morphology (white arrows). (iii) In the presence of DAPT and FGF2, CE cells ceased to proliferate and did not adopt a neural morphology. However under these conditions a greater proportion of Müller stem cells adopted a neural-like morphology. (Scale bar represents 100 μm.) (B) Western blotting of cell lysates revealed that CE cell preparations cultured under differentiating conditions (CE 6165, CE 6213 and CE 6334) did not express BRN3B, a transcription factor present in post-mitotic retinal ganglion cell precursors. However, increased expression of this protein was observed in Müller stem cells (MIO-M2, MIO-M5 and MIO-M7) cultured under similar conditions. (C) Examination of mRNA for photoreceptor gene expression revealed that in the presence of FGF2 non-pigmented CE cells and Müller stem cells expressed *NRL* and Rhodopsin, but that only Müller cells expressed S-Opsin. (CE = Ciliary Epithelium, FGF2 = Fibroblast Growth Factor 2.)

To compare the potential of spontaneously immortalized Müller glia and non-pigmented CE cells to differentiate into retinal neurons, cells were cultured at low density on Matrigel in the presence of FGF2. These conditions have been previously shown to promote the differentiation of Müller stem cells(Lawrence et al., 2007). In addition cells were also cultured in the presence of FGF2 and DAPT, an inhibitor of the Notch pathway which inhibits progenitor cell differentiation during development (Nelson et al., 2007). Following 7 days in culture, non-pigmented CE cells did not show any real change in morphology (Fig. 6A,B). When DAPT was added to these cells in culture, the rate of proliferation was markedly reduced, but cells did not extend neuronal processes (Fig. 6C). In contrast, a large proportion of spontaneously immortalized Müller glia cultured under similar conditions for 7 days adopted a neural morphology in the presence of FGF2 (Fig. 6E). Presence of DAPT caused a greater number of Müller glia to adopt neural morphology and extend long axon-like processes (Fig. 6F). Analysis of protein expression revealed that under differentiating conditions, three different preparations of immortalized Müller glia expressed BRN3B, a transcription factor expressed by post-mitotic ganglion cell precursors (Fig. 6B). However, three different preparations of CE cells did not express this protein under any of the conditions investigated. Investigation of mRNA expression revealed that both CE and immortalized Müller glia expressed the genes coding for the rod photoreceptor markers *NRL* and rhodopsin when cultured with FGF2 (Fig. 6C). These factors are also expressed *in situ* by cells of the human CE (Bertazolli-Filho et al., 2001). However, CE cells did not express genes for S-Opsin, which was observed in Müller glia cultured under similar conditions.

## 4 Discussion

This study shows that profound differences exist between ciliary epithelium and Müller glia with stem cell characteristics from the human eye, both of which have been shown to exhibit neural progenitor characteristics. Although the ciliary body contains pigmented and non-pigmented cells, these cells have not been previously dissociated to assess their proliferative and neurogenic properties. Several studies have reported that progenitor cells can be isolated from the pigmented layer of the ciliary epithelium of rats and mice (Tropepe et al., 2000; Das et al., 2005, 2006; Giordano et al., 2007). Although progenitor cells have been isolated from the ciliary body in larger mammals including pigs, non-human primates and humans, there have been no objective studies that identify whether cells reported to be progenitor cells are of pigmented or non-pigmented origin (Gu et al., 2007; MacNeil et al., 2007). Some reports have suggested that retinal stem cells are rare pigmented ciliary epithelial cells (Xu et al., 2007) and other studies in primates and humans have suggested that a stem cell population may exist in the non-pigmented CE (Fischer et al., 2001; Mayer et al., 2003), implying that differences may exist between the ciliary epithelium of rodents and humans, which may be important to identify when considering the potential of these cells to develop therapies using adult stem cells from the human eye.

To address these issues, we dissociated pigmented from non-pigmented CE cells from the human eye and cultured them under various conditions. Pigmented CE cells consistently failed to proliferate when cultured either as floating spheres or as monolayers. In contrast, non-pigmented CE cells proliferated when cultured as primary cultures in suspension or under adherent conditions either as primary or secondary cultures. In contrast, Müller glia that became spontaneously immortalized and that display stem cell characteristics did not proliferate when cultured in suspension, and when cultured under adherent conditions, they started forming small colonies after 2–3 weeks and did not depend on the presence of EGF for long term survival and proliferation. This may explain why many attempts at stem cell isolation from the adult human retina have been unsuccessful as most studies have attempted to establish primary cultures of these cells under non-adherent conditions (Tropepe et al., 2000; MacNeil et al., 2007). Non-pigmented CE cells proliferate faster during early passages when compared to Müller stem cells. However they proliferate for a long time in culture only when they had been initially cultured as spheres in suspension before adherence to plates. In the presence of EGF they proliferated for up to 22 passages investigated. This suggests that sphere formation may be necessary to de-differentiate these cells before they can be expanded in culture in the presence of EGF. This is supported by evidence that epithelial cells, such as RPE (Engelhardt et al., 2005) and limbal epithelial stem cells (Zhao et al., 2008) de-differentiate and acquire neuronal properties upon initial culture in suspension.

Based on the evidence presented in this study and several *in vivo* studies in small vertebrates (Fischer and Reh, 2001; Ooto et al., 2004; Karl et al., 2008), it is possible to suggest that the population of Müller glia isolated from the adult human retina which grow indefinitely *in vitro* and express markers of neural progenitors constitute true retinal stem cells. In common with adult neural stem cells from the brain (Goldman, 2003), immortal Müller cells possess a glial phenotype and express CRALBP, Vimentin and CD44, which is in accordance with previous studies that Müller glia and retinal neurons arise from a common multipotent retinal progenitor cell (Turner and Cepko, 1987). Since Müller glia are the last type of cell to be generated in the retina, it is possible that a sub-population of these cells may retain retinal progenitor characteristics, and could therefore de-differentiate, re-enter the cell cycle and form new retinal neurons. Although these cells have shown neurogenic properties in the early postnatal retina of chicks and rodents (Fischer and Reh, 2001; Ooto et al., 2004; Karl et al., 2008), no evidence for *in vivo* neurogenesis has yet been observed in humans despite the presence of these cells in the neural retina. Nevertheless, evidence for the presence of Müller glia with stem cell characteristics in the adult human retina provides a theoretical basis for the potential development of endogenous repair strategies.

That human Müller stem cells in culture expressed mRNA coding for ciliary epithelium cell markers, it may reflect that observed *in situ* where they form adherence junctions with the outer segments of photoreceptors (Tserentsoodol et al., 1998). A sub-population of hMSC also expressed RPE65, thought to be selectively expressed by RPE and cone photoreceptors. This raises the possibility that RPE65 may be involved in retinoid processing by Müller glia, supported by evidence that these cells are able to process retinoids (Kanan et al., 2008). Human Müller stem cells in culture also expressed mRNA coding for the transcription factor *NRL*, a rod photoreceptor gene, as well as Rhodopsin and S-Opsin, well characterized photoreceptor markers. These observations, together with the evidence of RPE65 expression, suggest that by expressing these genes Müller glia has the potential to differentiate into mature photoreceptors.

Non-pigmented CE cells expressed a higher level of pigmented epithelial mRNA markers compared to Müller glia, and were found to stain strongly for cytokeratins reflecting their phenotypic epithelial origin. Although non-pigmented CE cells expressed mRNA markers for neural progenitors, they did not express the corresponding proteins as seen with Müller glia. Under conditions known to promote the differentiation of immortalized Müller glia into retinal neurons, CE cells failed to adopt a neural morphology and did not express markers of ganglion cells, although they expressed some mRNA transcripts present in developing rod photoreceptors. These results confirm a previous demonstration that CE cells may not constitute true retinal stem cells (Cicero et al., 2009).

In contrast to other studies that reported positive staining for Nestin and SOX2 in CE cells (Goldman, 2003; Coles et al., 2004), we did not detect Nestin protein expression by CE cells and only a small minority of these stain for SOX2. However, no other studies have correlated immunostaining with gene expression, and many reports have shown that spheres may non-specifically stain for various factors and this may account for these differences. In addition, it has been suggested that neurosphere culture may affect cell proliferation and the positional cues to which cells are exposed (Jensen and Parmar, 2006), thus accounting for the possibility of non-specific staining previously reported by CE spheres.

From these observations, it is reasonable to conclude that CE cells have limited proliferative ability and lack true neurogenic potential. On this basis we believe that these cells do not constitute a suitable cell population for developing therapies to treat retinal disease. In contrast, Müller glia that become spontaneously immortalized and that exhibit stem cell properties *in vitro* may constitute a potential source of retinal neurons and fulfil the characteristics of true stem cells due to their proliferative and neurogenic ability. These cells may therefore constitute a viable target to develop therapeutic strategies by either cell transplantation or by induction of endogenous repair mechanisms.

## Figures and Tables

**Table 1:** Primer Sequences used for RT-PCR to assess gene expression of markers of retinal progenitors and mature retinal neurons by hMSC and non-pigmented CE cells.

Name	Forward sequence	Reverse sequence	cDNA location (base pairs)	Product Size (base pairs)
CRALBP	CGTGGCGGAGAGGGTGCAAG	GGTGCAGCGGACAGCCTCTG	360–528	169
CHX10	AGCTAGAGGAGCTGGAGAAG	CATGATGCCATCCTTGGCTG	556–846	258
GAPDH	CCACCCATGGCAAATTCCATGGCA	TCTAGACGGCAGGTCAGGTCCACC	188–785	598
MERTK	GGGAGATCGAGGAGTTTCTC	CGGCCTTGGCGGTAATAATC	2008–2325	388
MITF-A	TGAAGAGCCCAAAACCTATTACGA	GATCAATCAAGTTTCCCGAGACAG	170–906	736
NOTCH1	GCTGGACTGGTGAGGACTG	AGCCCTCGTTACAGGGGTT	977–1153	177
NRL	GGCTCCACACCTTACAGCTC	GGCCCATCAACAGGGACTG	118–311	212
PAX6	AGATGAGGCTCAAATGCGAC	GTTGGTAGACACTGGTGCTG	1102–1385	302
Rhodopsin	GCTTCCCCATCAACTTCCTCA	AGTATCCATGCAGAGAGGTGTAG	152–285	156
RPE65	GCCCAGGAGCAGGACAAAAG	GCGCATCTGCAAGTTAAAACCA	1529–1775	247
SHH	TGCTGCTGCTGGCGAGATGT	AATCGCTCGGAGTTTCTGGTG	153–369	217
S-Opsin	TAGCAGGTCTGGTTACAGGATG	GAGACGCCAATACCAATGGTC	347–474	148
SOX2	GGCAGCTACAGCATGATGC	TCGGACTTGACCACCGAAC	932–1149	236
ZO-1	CCAGAATCTCGGAAAAGTGC	ACCGTGTAATGGCAGACTCC	2736–23085	397

## References

[bib1] Ahmad I., Tang L., Pham H. (2000). Identification of neural progenitors in the adult mammalian eye. Biochem. Biophys. Res. Commun..

[bib2] Bertazolli-Filho R., Ghosh S., Huang W., Wollmann G., Coca-Prados M. (2001). Molecular evidence that human ocular ciliary epithelium expresses components involved in phototransduction. Biochem. Biophys. Res. Commun..

[bib3] Cicero S.A., Johnson D., Reyntjens S., Frase S., Connell S., Chow L.M., Baker S.J., Sorrentino B.P., Dyer M.A. (2009). Cells previously identified as retinal stem cells are pigmented ciliary epithelial cells. Proc. Natl. Acad. Sci. USA.

[bib4] Coles B.L., Angenieux B., Inoue T., Del Rio-Tsonis K., Spence J.R., McInnes R.R., Arsenijevic Y., van der K.D. (2004). Facile isolation and the characterization of human retinal stem cells. Proc. Natl. Acad. Sci. USA.

[bib5] Das A.V., James J., Rahnenfuhrer J., Thoreson W.B., Bhattacharya S., Zhao X., Ahmad I. (2005). Retinal properties and potential of the adult mammalian ciliary epithelium stem cells. Vis. Res..

[bib6] Das A.V., Zhao X., James J., Kim M., Cowan K.H., Ahmad I. (2006). Neural stem cells in the adult ciliary epithelium express GFAP and are regulated by Wnt signaling. Biochem. Biophys. Res. Commun..

[bib7] Engelhardt M., Bogdahn U., Aigner L. (2005). Adult retinal pigment epithelium cells express neural progenitor properties and the neuronal precursor protein doublecortin. Brain Res..

[bib8] Fimbel S.M., Montgomery J.E., Burket C.T., Hyde D.R. (2007). Regeneration of inner retinal neurons after intravitreal injection of ouabain in zebrafish. J. Neurosci..

[bib9] Fischer A.J., Hendrickson A., Reh T.A. (2001). Immunocytochemical characterization of cysts in the peripheral retina and pars plana of the adult primate. Invest. Ophthalmol. Vis. Sci..

[bib10] Fischer A.J., Reh T.A. (2001). Muller glia are a potential source of neural regeneration in the postnatal chicken retina. Nat. Neurosci..

[bib11] Fischer A.J., Reh T.A. (2003). Growth factors induce neurogenesis in the ciliary body. Dev. Biol..

[bib12] Giordano F., De Marzo A., Vetrini F., Marigo V. (2007). Fibroblast growth factor and epidermal growth factor differently affect differentiation of murine retinal stem cells in vitro. Mol. Vis..

[bib13] Goldman S. (2003). Glia as neural progenitor cells. Trends Neurosci..

[bib14] Gu P., Harwood L.J., Zhang X., Wylie M., Curry W.J., Cogliati T. (2007). Isolation of retinal progenitor and stem cells from the porcine eye. Mol. Vis..

[bib15] Gualdoni S., Baron M., Lakowski J., Decembrini S., Smith A.J., Pearson R.A., Ali R.R., Sowden J.C. (2010). Adult ciliary epithelial cells, previously identified as retinal stem cells with potential for retinal repair, fail to differentiate into new rod photoreceptors. Stem Cells.

[bib16] Inoue T., Coles B.L., Dorval K., Bremner R., Bessho Y., Kageyama R., Hino S., Matsuoka M., Craft C.M., McLnnes R.R., Temblay F., Prusky G.T., van der Kooy D. (2009). Maximizing functional photoreceptor differentiation from adult human retinal stem cells. Stem Cells.

[bib17] Jensen J.B., Parmar M. (2006). Strengths and limitations of the neurosphere culture system. Mol. Neurobiol..

[bib18] Kanan Y., Kasus-Jacobi A., Moiseyev G., Sawyer K., Ma J.X., Al-Ubaidi M.R. (2008). Retinoid processing in cone and Muller cell lines. Exp. Eye Res..

[bib19] Karl M.O., Hayes S., Nelson B.R., Tan K., Buckingham B., Reh T.A. (2008). Stimulation of neural regeneration in the mouse retina. Proc. Natl. Acad. Sci. USA.

[bib20] Klassen H., Ziaeian B., Kirov, Young M.J., Schwartz P.H. (2004). Isolation of retinal progenitor cells from post-mortem human tissue and comparison with autologous brain progenitors. J. Neurosci. Res..

[bib21] Lawrence J.M., Singhal S., Bhatia B., Keegan D.J., Reh T.A., Luthert P.J., Khaw P.T., Limb G.A. (2007). MIO-M1 cells and similar muller glial cell lines derived from adult human retina exhibit neural stem cell characteristics 1. Stem Cells.

[bib22] Limb G.A., Salt T.E., Munro P.M., Moss S.E., Khaw P.T. (2002). In vitro characterization of a spontaneously immortalized human Muller cell line (MIO-M1). Invest. Ophthalmol. Vis. Sci..

[bib23] MacNeil A., Pearson R.A., MacLaren R.E., Smith A.J., Sowden J.C., Ali R.R. (2007). Comparative analysis of progenitor cells isolated from the iris, pars plana, and ciliary body of the adult porcine eye. Stem Cells.

[bib24] Mayer E.J., Hughes E.H., Carter D.A., Dick A.D. (2003). Nestin positive cells in adult human retina and in epiretinal membranes. Br. J. Ophthalmol..

[bib25] Moe M.C., Kolberg R.S., Sandberg C., Vik-Mo E., Olstorn H., Varghese M., Langmoen I.A., Nicolaissen B. (2009). A comparison of epithelial and neural properties in progenitor cells derived from the adult human ciliary body and brain. Exp. Eye Res..

[bib26] Nelson B.R., Hartman B.H., Georgi S.A., Lan M.S., Reh T.A. (2007). Transient inactivation of Notch signaling synchronizes differentiation of neural progenitor cells. Dev. Biol..

[bib27] Nishina S., Hirakata A., Hida T., Sawa H., Azuma N. (1997). CD44 expression in the developing human retina. Graefes Arch. Clin. Exp. Ophthalmol..

[bib28] Ooto S., Akagi T., Kageyama R., Akita J., Mandai M., Honda Y., Takahashi M. (2004). Potential for neural regeneration after neurotoxic injury in the adult mammalian retina. Proc. Natl. Acad. Sci. USA.

[bib29] Raymond P.A., Barthel L.K., Bernardos R.L., Perkowski J.J. (2006). Molecular characterization of retinal stem cells and their niches in adult zebrafish. BMC Dev. Biol..

[bib30] Tropepe V., Coles B.L., Chiasson B.J., Horsford D.J., Elia A.J., McInnes R.R., van der Kooy D. (2000). Retinal stem cells in the adult mammalian eye. Science.

[bib31] Tserentsoodol N., Shin B.C., Suzuki T., Takata K. (1998). Colocalization of tight junction proteins, occludin and ZO-1, and glucose transporter GLUT1 in cells of the blood-ocular barrier in the mouse eye. Histochem. Cell Biol..

[bib32] Turner D.L., Cepko C.L. (1987). A common progenitor for neurons and glia persists in rat retina late in development. Nature.

[bib33] Wan J., Zheng H., Xiao H.L., She Z.J., Zhou G.M. (2007). Sonic hedgehog promotes stem-cell potential of Muller glia in the mammalian retina. Biochem. Biophys. Res. Commun..

[bib35] Xu S., Sunderland M.E., Coles B.L., Kam A., Holowacz T., Ashery-Padan R., Marquardt T., McInnes R.R., van der Kooy D. (2007). The prolifeartion and expansion of retinal stem cells require functional Pax6. Dev. Biol..

[bib34] Zhao X., Das A.V., Bhattacharya S., Thoreson W.B., Sierra J.R., Mallya K.B., Ahmad I. (2008). Derivation of neurons with functional properties from adult limbal epithelium: implications in autologous cell therapy for photoreceptor degeneration. Stem Cells.

